# Comparison of biological H_2_S removal characteristics between a composite packing material with and without functional microorganisms

**DOI:** 10.1038/srep42241

**Published:** 2017-02-13

**Authors:** Rencheng Zhu, Shunyi Li, Xiaofeng Bao, Éric Dumont

**Affiliations:** 1 School of Chemical Engineering and Energy, Zhengzhou University, Zhengzhou, 450001, China; 2 Atmospheric Environment Institute, Chinese Research Academy of Environmental Sciences, Beijing, 100012, China; 3 Department of Energy Systems and Environment, UMR CNRS 6144 GEPEA, École des Mines de Nantes, Nantes, 44307, France

## Abstract

The performances of two identical biofilters, filled with a new composite packing material (named CM-5) embedded with functional microorganisms or sterilized CM-5 without microorganisms, were investigated for H_2_S treatment. Running parameters in terms of microbial counts, pressure drops, and inlet and outlet H_2_S concentrations were measured. The results show that the microbial count of the CM-5 was approximately ×10^5^ CFU/g before being filled into the biofilter, while that of the sterilized CM-5 was negligible. The functional microorganisms embedded in CM-5 adapted to the environment containing H_2_S quickly. In most cases, pressure drops of the CM-5 biofilter were slightly higher than those of the sterilized CM-5 biofilter when the gas flow rate was 0.6–2.5 m^3^/h. The maximum elimination capacity (EC) of the CM-5 biofilter in treating H_2_S could reach up to 65 g/(m^3^·h) when the loading rate (LR) was approximately 80 g/(m^3^·h). If the LR was much higher, the measured EC showed a slight downward trend. The experimental ECs of biofilters were fitted by two typical dynamic models: the Michaelis-Menten model and the Haldane model. Compared with the Michaelis-Menten model, the Haldane model fit the experimental ECs better for the two biofilters because of the presence of the substrate inhibition behaviour.

It is widely known that malodorous hydrogen sulfide (H_2_S) emissions have potential extreme toxicity with respect to human health and aggressive corrosion with respect to buildings^1^,^2^. Considerable amounts of H_2_S are emitted from wastewater treatment and industrial activities, such as paper manufacturing and rubber processing^3^. Except for traditional physico-chemical technologies, H_2_S emissions can also be removed using biotechnologies, such as biofiltration. Biofiltration is considered to be good for the environment because it consumes lower energy and generates little undesirable byproducts during treatment. Moreover, other intermediate products, such as HS^−^ and S^2−^, could be used as energy sources by microorganisms^4^.

Microorganisms play a major role in the biofiltration system because the purification efficiency mainly depends on the microbial activity. However, the core of the biofiltration system might be the packing materials. A suitable packing material could provide an ideal microenvironment for microorganism growth, such as abundant nutrients and uniform distributions of water and air^5^,^6^,^7^. According to previous studies, the optimal packing materials should have specific physicochemical properties, such as suitable particle size, large surface area, high porosity, good mechanical resistance and moisture retention. Moreover, a high buffer capacity to avoid large pH fluctuations and abundant nutrient sources are also necessary^4^,^8^,^9^,^10^,^11^. Some studies have also shown that optimal packing material should contain microorganisms to shorten the period of biofilm formation at start-up^12^,^13^,^14^.

A plethora of research on different types of packing materials (organic, inorganic and synthetic materials) has been conducted for biofilters^15^. Organic materials, such as waste compost, sugarcane bagasse, coconut fibre and wood bark, have advantages in terms of nutrient sources and native microorganisms, while they tend to compact after running a course of time, which will result in increasing pressure drops and cause inhomogeneous air flow distributions^16^. Inorganic materials, such as lava rock, metal oxides, ceramsite and pall ring, can also be used. However, these inorganic materials must be inoculated with microorganisms with a period of 7–14 days. Moreover, inorganic packing materials cannot provide nutrients for microorganisms. To overcome the inherent problems of both organic and inorganic materials, such as bed compaction or lack of nutrients, synthetic packing materials have attracted researchers’ attention. Development of new composite packing materials has become a new research direction. Over the last few years, the formulation and performance of several synthetic packing materials have been reported. For example, Chan, W. *et al*.^17^ synthesized a composite bead containing poly/peat/KNO_3_, which could remove 98% of volatile organic compounds (VOCs) in a biofilter. Dumont, E. *et al*.^15^ developed a synthetic material (named UP20); this material not only supplies nutrients but also has good pH buffering capacity. A composite material for biofiltration was prepared by JiaDe, W. *et al*.^18^; this novel media has a function of slow nutrient release. However, only few papers have evaluated the performance of packing material embedded functional microorganisms in a biofilter^12^,^19^.

A recent study highlighted that a composite packing material (called CM-5, representing a composite material embedding more than ×10^5^ CFU/g functional microorganisms) is a good packing material in terms of embedding functional microorganisms, providing nutrients and buffering pH fluctuation^12^. The aim of this study is to further investigate the contribution of functional microorganisms embedded in the materials. A crucial comparison was carried out using two identical biofilters filled with original CM-5 (i.e., the materials embedded with microorganisms) and sterilized CM-5 (i.e., the material without microorganisms), respectively. During the manufacturing process, the possibility of shutdown due to mechanical failures should be considered. Therefore, one week of interruption was set in the test to investigate the response of the composite packing materials.

## Results and Discussion

### Microbial counts

**Figure 1:**
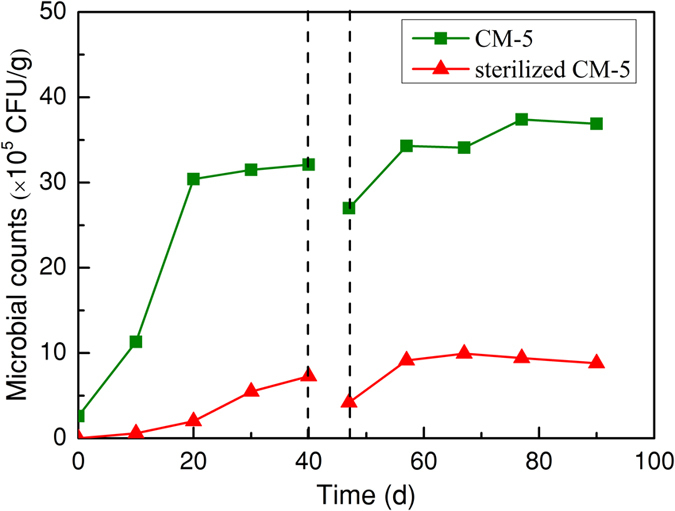
Microbial counts versus time for CM-5 and sterilized CM-5, respectively.

Figure 1 shows the microbial count behaviour of CM-5 and sterilized CM-5 biofilters along with the operation. There were approximately ×10^5^ CFU/g of CM-5 at the beginning of the experiment. After running 10 days, the quantity of microorganisms on CM-5 reached ×10^6^ CFU/g, indicating that the functional microbial communities adapted to the environment containing H_2_S and propagated rapidly. In contrast, the microbial quantity on the sterilized CM-5 was small and the breed rate was slow. Evidently, the quantity of microorganisms on CM-5 was much higher than that of the sterilized CM-5 throughout the experiments. One reason was that there were large amounts of functional microorganisms on CM-5, while those of sterilized CM-5 were mainly from waste gas and spraying water. Another speculation might be that the bioavailability of nutrients contained in sterilized CM-5 decreased through the sterilization process. Because of mechanical failure or other incidents, temporary interruption of the bio-treatment system is unavoidable. To investigate the impact of interruption on the microbial behaviour in a biofilter, a seven-day interruption was set at the 40^th^ day. Figure 1 shows that both biofilters were impacted by the interruption and that their microbial quantities experienced some decrease. This result was mainly attributed to the moisture of the packing bed being too low, leading to some microorganisms being dormant or dying during the interruption. However, after resumption, quantities of microorganisms on both CM-5 and sterilized CM-5 recovered rapidly to ×10^6^ and ×10^5^ CFU/g of material, respectively. The short recovery period might be due to the rapid recuperation of enzymatic activity and microorganism metabolism from dormancy^11^. This phenomenon was consistent with that obtained in some studies. For example, Moe, W. *et al*.^20^ observed that a five-day period was needed to recover after a two-day shutdown; Maestre, J. *et al*.^11^ reported that the recovery period was 3–7 days after a five-day shutdown. This result indicated that both CM-5 and sterilized CM-5 could shorten the start-up period and limit the negative effects due to shutdowns and loading rate fluctuations, which might be mainly attributed to their nutrient slow-release capacity.

Overall, CM-5 contained abundant microorganisms, which proliferated rapidly after introducing a mixture gas including H_2_S. Microbial counts of both CM-5 and sterilized CM-5 decreased during the interruption period, but they recovered rapidly after re-starting the system.

### Pressure drops

**Figure 2:**
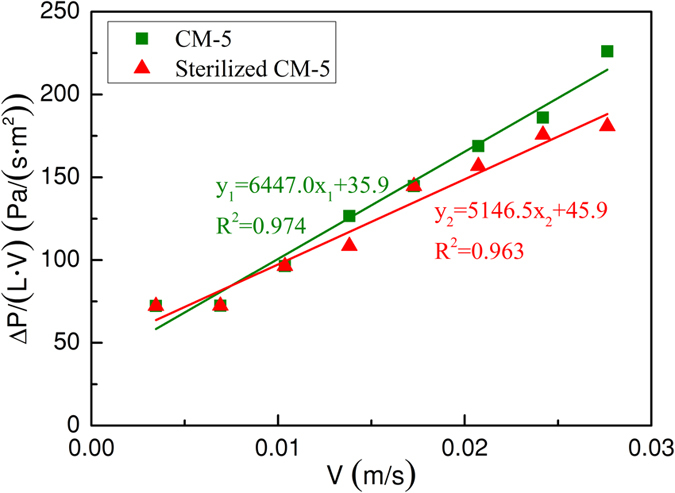
Average pressure drop values for CM-5 and sterilized CM-5 at the 30th day.

The pressure drops are closely related to the operating cost of the biofilter system, which could be impacted by many factors, such as the water content, airflow velocity, packing material size and material shape. In this section, only the superficial gas velocity and microorganisms on the materials were considered. Figure 2 shows the relationship between pressure drop values and superficial gas velocity after the two biofilters were run for 30 days. The results reveal that the term ΔP/(L·V) (ΔP is the pressure drop along the packing material bed length (Pa); L is the height of the packing material layer (m); V is the superficial air velocity (m·s^−1^)) was proportional to the gas velocity, indicating that pressure drop values fit the Ergun equation very well. The linear correlations were 0.974 and 0.963 for the CM-5 biofilter and sterilized CM-5 biofilter, respectively. Different amounts of increase in pressure drops were observed for the two packing materials. Specially, the β constant values, representing the energy losses, calculated from the fitted equations were 6477.0 and 5146.5 Pa/(s^2^·m^3^) for CM-5 and sterilized CM-5, respectively. Clearly, the β constant value (β (Pa·s^2^/m^3^) is the regression parameter) of CM-5 was higher than that of sterilized CM-5, which was attributed to the biomass on CM-5 being higher than that on sterilized CM-5, as shown in Fig. 1. More biomass on the material surface might result in the void space among the CM-5 becoming a little smaller, increasing the pressure.

The β value of CM-5 was similar to that of the synthetic nutritional material (UP20) (8,440 Pa/(s^2^·m^3^))^15^ and significantly lower compared with those obtained for polyurethane foam, sugarcane bagasse and coconut fibre: 55,813, 46,708, 114,775 Pa/(s^2^·m^3^), respectively^1^. It should be noted that CM-5 and UP20 have a similar shape. The comparison also illustrated that the material had significant impacts on the pressure drops. According to the experimental measurements, the biggest pressure drop value was 6.3 Pa/m for a gas velocity of approximately 0.028 m/s (i.e., 100 m/h) for CM-5 and sterilized CM-5. For similar gas velocity, pressure drop values reported in the literature were approximately 20 Pa/m for expanded schist^15^ and 40 Pa/m for peat^4^, which may be because the size of CM-5 was larger than that of UP20 and peat, which would generate larger gaps and lead to smaller air pressure.

Overall, pressure drop of the biofiltration increased as the gas velocity increased, and the pressure values suited the Ergun equation quite well. In most cases, the pressure drop values of CM-5 were slightly higher than those of sterilized CM-5.

### Loading rate and elimination capacity

**Figure 3:**
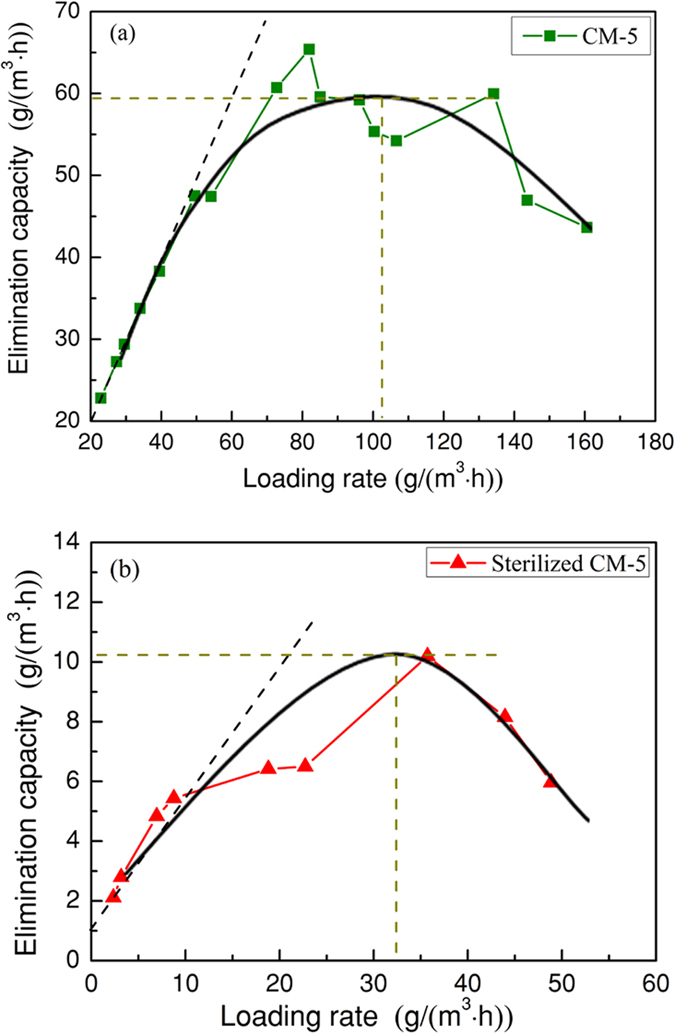
Impacts of the inlet H_2_S loading rate on the elimination capacity for the CM-5 biofilter (**a**) and sterilized CM-5 biofilter (**b**): EBRT = 48 s.

Loading rate (LR) of the pollutant always fluctuates during the actual operation, which may impact the elimination capacity (EC). In this study, the relationship between LR and EC of H_2_S was investigated; the LR was controlled by changing the inlet H_2_S concentration and keeping the inlet gas flow rate constant. Figure 3 shows the variation trend of EC with increasing LC for the CM-5 and sterilized CM-5 biofilters. Clearly, the response of the dependent variable (EC) to the variable (LR) was similar for CM-5 and sterilized CM-5 biofilters. ECs of both CM-5 and sterilized CM-5 increased to a maximum and then decreased slightly as LRs increased. Take CM-5 for example; the removal efficiency (RE) of the CM-5 biofilter was approximately 100% when LR was lower than 49.5 g/(m^3^·h), then RE gradually decreased. As LR increased, EC increased first and reached a peak: approximately 60 g/(m^3^·h). When the LR was higher than 80 g/(m^3^·h), EC was found to drop noticeably. Additionally, the EC of sterilized CM-5 reached the maximum value (10.2 g/(m^3^/h)) when LR was approximately 35 g/(m^3^/h). Kim, J. *et al*.^21^ observed similar result in treating H_2_S by using biomedia encapsulated by polyvinyl alcohol and sodium alginate. The reason may be that the biological growth was inhibited by the high concentration of H_2_S^17^. Compared with Fig. 3(a and b), it can be found that the measured maximum EC of the CM-5 biofilter was much higher than that of the sterilized CM-5 biofilter. This result was mainly attributed to the functional microorganism communities because other conditions of the two biofilters were almost the same.

Overall, as the loading rate increased, the elimination capacities of CM-5 increased first and then showed a slight downward tendency. The ECs of CM-5 were much higher than those of sterilized CM-5.

### Biodegradation kinetics

The pollutant elimination is mostly closely related to the microbial activity on the packing material surface, which could be described using biodegradation kinetics. The modified Michaelis-Menten model is commonly used to describe the relationship between the elimination behaviour and substrate concentration. This model mainly contains two reaction phases: first-order reaction and zero-order reaction. When the EC presents inhibition, a Haldane model containing a substrate inhibition term might be used to describe the elimination behaviour^22^. EC_max_ could be deduced from K_s_, EC′, 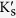 and K_i_, and these parameters could be calculated via the regressions of C_in_, C_out_, LR and EC according to eqs (1) and (2)^22^ (definitions of K_s_, EC′, 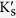, K_i_, C_in_, C_out_, LR and EC can be found in Supplementary Table S1):


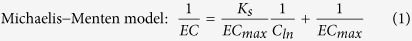



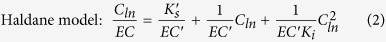


**Figure 4:**
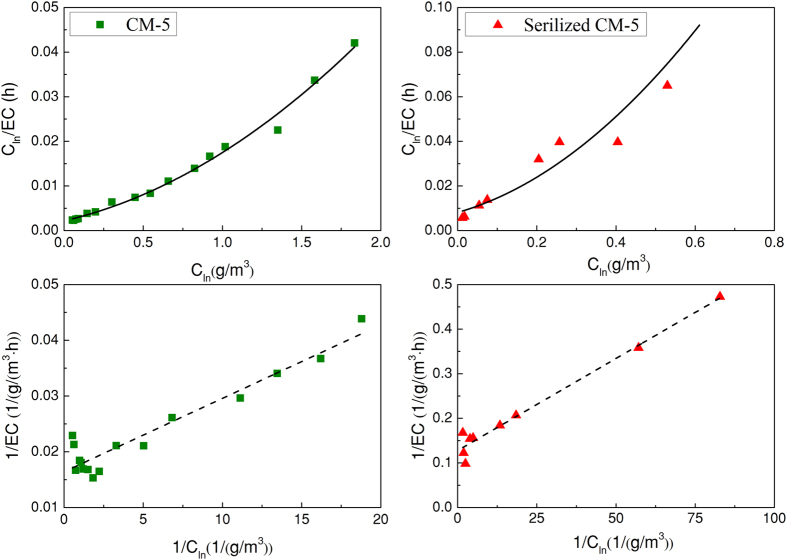
Experimental points and kinetics models: black line for the Haldane model and dashed line for the Michaelis-Menten model.

Figure 4 shows the fitting results with the Haldane model and Michaelis-Menten model, respectively, based on data points of the CM-5 and sterilized CM-5 biofilters. Table 1 summarizes the biodegradation kinetics values calculated from the two models. The results show that the Michaelis-Menten model fit the experimental data satisfactorily, while the Haldane model fit the CM-5 biofilter data better because the Michaelis-Menten model does not consider the inhibition of the substrate. Actually, the high H_2_S concentration in waste gas would poison the microorganisms and decrease the purification efficiency. The result was partial in line with some previous literature^22^,^23^. As shown in Table 1, comparing EC_max_ based on the Michaelis-Menten model with that based on the Haldane model, it can be found that EC_max_ based on the Haldane model was slightly lower than that based on the Michaelis-Menten model for the two biofilters. It could be considered that there was a slight inhibition effect of the substrate on the microbial population for both CM-5 and sterilized CM-5 biofilters. Figure 3 also verified the substrate inhibition phenomenon, in which EC decreased when LR was higher than 80 g/(m^3^·h) and 30 g/(m^3^·h) for the CM-5 and sterilized CM-5 biofilters, respectively.

In this study, EC_max_ obtained from the two models were approximately 62 and 8 g/(m^3^·h) for the CM-5 biofilter and sterilized CM-5 biofilter, respectively. The EC_max_ value of CM-5 was significantly higher than those reported in previous studies, as listed in Table 2. The EC_max_ values of CM-5 and sterilized CM-5 obtained in this experiment were low in comparison with other studies with polyurethane foam, sugarcane bagasse and coconut fibre^1^ but were compatible with results from mixed microbial population studies reported in the literature^22^. According to the report^7^, the rates of many industrial emissions were approximately 10–45 g/(m^3^·h) in production processes. Therefore, it could be inferred that the packing material of CM-5 might be applied in actual industrial operation. The packing bed could be used in modular format, allowing the use of as many modules as necessary so that the outlet gas could achieve a minimum concentration under the condition of permission pressure drop.

Overall, there were slight inhibition effects of H_2_S on both the CM-5 and sterilized CM-5 biofilters during the test. The Haldane model fit the experimental data better than the Michaelis-Menten model. The calculated maximum ECs of the CM-5 biofilter were much higher than those of the sterilized CM-5 biofilter.

## Methods

### Packing materials preparation

The CM-5 with functional microorganism communities and the sterilized CM-5 without microorganisms were used to fill two identical biofilters, respectively. The CM-5 was self-developed in the laboratory using matured compost, porous perlite, cement, calcium carbonate (CaCO_3_), plant fibre (sieved from dry compost) and inorganic binder. The suitable proportions in mass of these raw materials were 17%, 18%, 18%, 13%, 7% and 27%, respectively. CaCO_3_ was used to inhibit large pH fluctuations and matured compost was used as the nutrient sources. The functional microbial communities embedded in the CM-5 were obtained from a biofilter filled with corncob materials, which was used to remove the waste gas containing H_2_S. More information regarding materials or substances used in the preparation of CM-5 can be found in our previous paper^12^. The sterilized CM-5 was obtained by placing CM-5 in an autoclave for high-pressure sterilization for 30 min at 121 °C. This material was columnar and its surface looked rough. The appearances of the original CM-5 and the sterilized CM-5 can be found in Supplementary Fig. S1. Some selected characteristics of the CM-5 compared with single and synthetic materials from other studies are presented in Table 3. Clearly, the physical properties were comparable with most of the reference materials, illustrating that CM-5 might be suitable for application in a biofilter.

### Experimental set-up

Two identical three-stage biofilters constructed using polyvinyl chloride (PVC) pipes were used in this study. The schematic diagram of the experimental system can be found in Supplementary Fig. S2. A 20-cm space above each packing layer was set to allow for air flow redistribution. The two biofilters were filled with 20.1 litres of CM-5 and sterilized CM-5, respectively. From bottom to top, the three sections were filled with packing materials to a height of 40 cm, 30 cm, and 30 cm, respectively. Four sampling ports were located along the wall of the biofilter for gas sampling and pressure measurements. Their locations were inlets, 15 cm above every packing layer, and outlets. Detailed information regarding the system can be found in our previous papers^12^,^19^. Operating conditions of the two biofilters are summarized in Table 4.

Mixed air was introduced at the bottom of the two biofilters directly. The H_2_S concentration, controlled by two gas flowmeters, was generated by mixing pure H_2_S from a gas cylinder and fresh air in a mix chamber. H_2_S concentrations were detected using a GT901-H_2_S device (accuracy: 0.1 ppm, Shenzhen, China). During the operation process, no biomass or nutritive solution was introduced into the biofilters artificially. The system was operated at room temperature throughout the experiment. To simulate the interruption of a biofilter occasionally occurring due to either mechanical or electrical failures in actual operation, a seven-day interruption was set at the 40^th^ day.

Moisture content of the packing materials was measured by taking the materials out randomly from the biofilter and placing them in an oven at 105 °C until the weight remained stable. More information regarding the moisture content measurement can be found in our previous paper^12^. The moisture content of the packing bed was controlled at a certain level (40–60%) by spraying tap water from the top. The leachate was collected at the bottom and recirculated to the top by a peristaltic pump. The circulating water was sprayed for 15 min (approximately 3 L) every day. Note that the spraying period was 15 min every 4 hour during the first day. The pH was not adjusted artificially during the experiment because the CM-5 contained alkaline matters, which could neutralize the acidity produced by H_2_S. No other microorganisms in the two biofilters were inoculated, except for the microorganisms embedded in the CM-5 and the native ones contained in the water or air. To determine the quantity of microorganisms forming on the packing materials, granules were sampled from each module of the running biofilter homogeneously. Microbial amounts were measured using the plate count method^12^.

Pressure drops were measured during normal operation under the condition of no spraying water. The pressure drop was detected using a Testo 510 device (accuracy: ±0.05 hPa, Testo, Germany) under different gas flow rates. To describe load loss behaviour and compare with other studies, experimental data were processed according to the Ergun eq. (3):


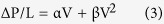


where ΔP is the pressure drop along the packing materials’ bed length (Pa); L is the height of the packing material layer (m); V is the superficial air velocity (m/s); α (Pa·s/m^2^) and β (Pa·s^2^/m^3^) are the regression parameters^23^.

Several terms used to evaluate the performance of biofilters and the definitions of some parameters used to fit experimental data with the Michaelis-Menten model and Haldane model are displayed in Supplementary Table S1^23^.

## Additional Information

**How to cite this article:** Zhu, R. *et al*. Comparison of biological H_2_S removal characteristics between a composite packing material with and without functional microorganisms. *Sci. Rep.* **7**, 42241; doi: 10.1038/srep42241 (2017).

**Publisher's note:** Springer Nature remains neutral with regard to jurisdictional claims in published maps and institutional affiliations.

## Supplementary Material

Supplementary Information

## Figures and Tables

**Table 1:** Biodegradation kinetics values determined from the Michaelis-Menten model (EC_max_ and *K*_*s*_) and the Haldane model (*EC*^*′*^, 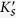, *K*_*i*_ and *EC*_*max*_).

Models	CM-5 biofilter	Sterilized CM-5 biofilter
Michaelis-Menten model	*EC*_*max*_ = 62.2	*EC*_*max*_ = 8.4
	*K*_*s*_ = 0.08	*K*_*s*_ = 0.03
Haldane model	*EC*′ = 122.3	*EC*′ = 19.83
	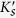 = 0.27	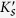 = 0.16
	*K*_*i*_ = 1.15	*K*_*i*_ = 0.35
	*EC*_*max*_ = 60.9	*EC*_*max*_ = 7.8

*EC*_*max*_, *EC*^*′*^: g/(m^3^·h); *K*_*s*_, 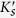, *K*_*i*_: g/m^3^.

**Table 2:** Maximum elimination capacities (g/(m^3^·h)) reported in previous papers.

Packing materials	EBRT(s)	Maximum value of EC (g/(m^3^ h))	References
Polyurethane foam	49	66.6	1
Coconut fibre	49	68.8	1
Sugarane bagasse	49	72.9	1
mature and sawdust	27	10–45	7
Schist	16	30	15
Peat	12	55	24
wool compost	25	36.1	25
Compost-PVC	60	21	26
Expanded schist	35	42	22
Peat-UP20	57	21	15
UP20 + expanded schist	19	36.4	27

**Table 3:** Some characteristics of CM-5 compared with other materials.

	Dimension	Bulk density	Moisture content	Specific surface area
	mm	Kg/m^3^	%	m^2^/g
Corncob^12^	10 × 10 × 15	110	70–80	0.22–0.24
Ceramsite^12^	10–13	420–460	<10	1.5–5
UP20^28^	Φ7 × 15	920	47	<1
PVA^17^	Φ4.0	692	50.5–66.8	—
BIOSORBENS^29^	5–25 (90%)	650	25	40.9
**CM-5**	**Φ12 × 20**	**470–472**	**47–52**	**3.28–3.91**

**Table 4:** Operating conditions of the two biofilters.

Parameter	Value
Inner diameter of column (mm)	160
Bulk density (kg/m^3^)	470
Bed porosity^a^	0.38
Volume of packing material (L)	20.1
Flow rate (m^3^/h)	0.6–2.5
EBRT (s)	29–121
Superficial velocity V (m/s)	0.008–0.035

^a^Bed porosity is calculated as follows: divide the bulk volume (20.1 L) by the void volume (subtracting the true volume from the bulk volume).
