# Supplementary material for: Comparison of biological H2S removal characteristics between a composite packing material with and without functional microorganisms
Source: Sci Rep. 2017 Feb 13;7:42241. doi: 10.1038/srep42241 (PMC5304181; doi:10.1038/srep42241)
Supplement: Supplementary Information [file srep42241-s1.pdf]

Appendix A. Supplementary Information:

Comparison of biological H_2S removal characteristics between a composite packing material with and without functional microorganisms

Rencheng Zhu[1], Shunyi Li[1, *], Xiaofeng Bao[2] & Éric Dumont[3]

[1] School of Chemical Engineering and Energy, Zhengzhou University, Zhengzhou, 450001, China
[2] Atmospheric Environment Institute, Chinese Research Academy of Environmental Sciences, Beijing, 100012, China.
[3] Department of Energy Systems and Environment, UMR CNRS 6144 GEPEA, École des Mines de Nantes, Nantes, 44307, France

* Current address: School of Chemical Engineering and Energy, Zhengzhou University, Zhengzhou, 450001, China.

Correspondence and requests for materials should be addressed to S.L. (lsy76@zzu.edu.cn) or R.Z. (zhurencheng2006@163.com)

4 pages

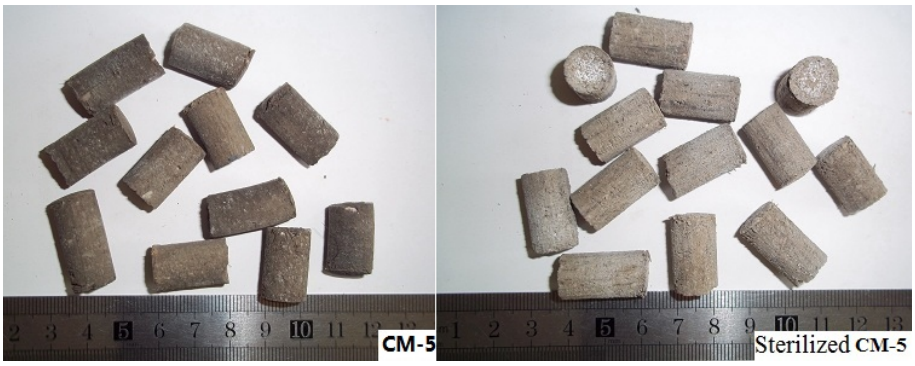

Figure S1. Packing materials: CM-5 and sterilized CM-5.

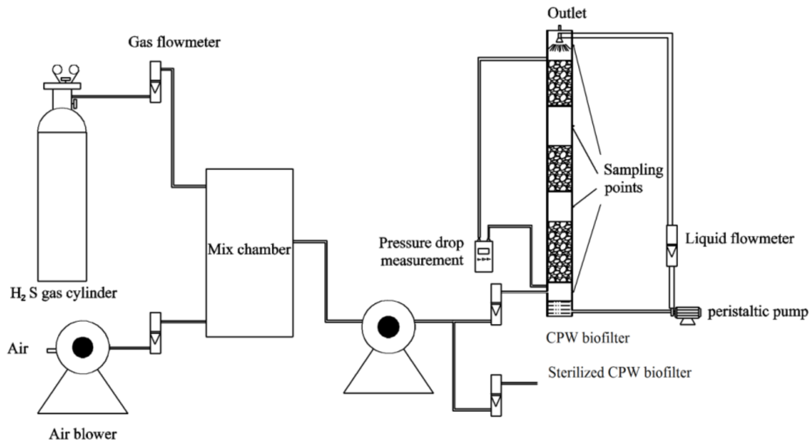

Figure S2. Schematic diagram of the experimental system for H_2S treatment.

Table S1. Definition of parameters and equations used in this study.

Terms	Definitions	Notes
Empty bed residence time (s)	$EBRT = \frac{V_f}{Q}$	
Loading rate (g/(m^3·h))	$LR = \frac{Q}{V_f} C_{in}$	V_f: packing material volume (m^3)<br>Q: gas flow rate (m^3/h)
Elimination capacity (g/(m^3·h))	$EC = \frac{Q}{V_f} (C_{in} - C_{out})$	C_{in}: inlet H_2S concentrations (g/m^3)<br>C_{out}: outlet H_2S concentrations (g/m^3)
Removal efficiency (%)	$RE = \frac{C_{in} - C_{out}}{C_{in}} \times 100$	
Michaelis-Menten model	$EC = \frac{EC_{max} C_{ln}}{K_s + C_{ln}}$<br>$C_{ln} = \frac{C_{in} - C_{out}}{\ln(C_{in}/C_{out})}$	EC_{max}: maximal elimination capacity (g/(m^3·h))<br>K_s: saturation constant (g/m^3)<br>C_{ln}: logarithmic average of the inlet and outlet H_2S concentration (g/m^3)
Haldane model	$EC = \frac{EC' C_{ln}}{K_s' + C_{ln} + (C_{ln}^2/K_i)}$<br>$EC_{max} = \frac{EC'}{1 + 2\sqrt{K_s'/K_i}}$	EC': maximal elimination capacity in the absence of inhibition (g/(m^3·h))<br>K_s': saturation constant (g/m^3)<br>K_i: inhibition constant (g/m^3)
